# Teaching pre-clinical medical students remotely in Nigeria post Covid-19 pandemic: can past experiences shape future directions?

**DOI:** 10.1186/s12909-024-05508-w

**Published:** 2024-05-09

**Authors:** Beatrice Emma-Okon, Michal Tombs, Rufus Akomolafe, Olugbenga Ayannuga

**Affiliations:** 1 Faculty of Basic Medical Sciences, Obafemi Awolowo University, Ile-Ife, Nigeria; 2 Centre for Medical Education, School of Medicine, Cardiff University, Cardiff, UK

**Keywords:** Pre-clinical, Online teaching, Covid − 19, Nigeria

## Abstract

**Purpose:**

Online teaching has gained popularity in recent years, but changes have been slower to implement in Lower or Medium Income Countries (LMIC). The aim of this research was to build upon educators’ experiences of remote teaching during Covid-19 to inform the development of a blended learning approach for teaching pre-clinical subjects at the Faculty of Biomedical Sciences at Obafemi Awolowo University, Ile-Ife, Nigeria (OAU).

**Methods:**

The Critical Incident Technique (CIT) was used in this exploratory study. Participants were invited to either complete an online qualitative questionnaire or take part in an online structured interview, which were hosted on Microsoft platforms. Data were obtained from eighteen educators and were analyzed using thematic analysis.

**Results:**

Findings suggest that most educators (72%) continued to engage with remote teaching post-pandemic. All lab-based practical topics returned to being in-person, and teachers’ experiences highlighted that a new blended learning approach should focus on asynchronized online teaching of didactic subjects. Five main themes captured educators’ experiences and lessons learned regarding online teaching including: skills and training, teachers’ motivation and attitudes, internet and connectivity, learners’ behaviors, and socio-economic constraints.

**Conclusion:**

Findings provided additional evidence on the way in which educators in LMIC would like to build upon the positive aspects of online teaching and move towards a blended learning model. However, the implementation of such an approach should consider students’ and faculty’s needs and socio-economic constraints.

## Introduction

The last decade has been characterized by an increase of remote teaching of pre-clinical subjects such as anatomy, biochemistry, and physiology across the globe [1–3]. Such changes have been slower to implement in Lower- or Middle-Income Countries (LMIC) and it was the abrupt shift to remote delivery that served as a catalyst for change [4]. The COVID-19 pandemic brought about significant changes in educational systems, and this was the situation in OAU College of Health Sciences. Pre-covid, the teaching of pre-clinical subjects was only conducted face to face as there were no institutional guidelines or facilities regarding online teaching. During lockdown, educators had to put emergency measures in place and migrated to remote teaching after attending a one-day rotational training.

Many institutions were able to quickly transition to online teaching during the pandemic because they had customized Learning Management Systems where instructional materials were hosted and the teaching process was facilitated (e.g., Moodle, Blackboard or Canvas) [5]. In developing countries, where such systems were not in place pre-pandemic, the transition to online teaching was more challenging [6]. This was the case at OAU, where quality of online teaching varied from one educator to another, and content was delivered via different platforms. This practice continued until lockdown measures had eased and educators and learners were able to return to classes. Educationalists were then faced with the question of what next, and whether and how online teaching should continue.

Studies conducted post-pandemic point towards an increased demand for online teaching and that the experience has brought about an openness towards innovation and new learning opportunities [7, 8]. The unintended gains propelled higher education institutions to revise their educational strategy towards a blended model [9] and OAU is now considering the use of a LMS, which is an important step forward towards a blended learning model.

The research question of this study was explorative in nature and focused on examining the experiences of educators to identify lessons learned that can inform future institutional directions regarding the teaching of pre-clinical subjects. The objectives were to: (I) ascertain the extent to which educators continued to teach online post-pandemic, and (II) examine enablers and challenges to online teaching and the lessons learned from these. This was in line with Adult Learning Theory that recognizes experience and perceptions of needs as a source of learning and development [10]. Context specific factors were explored as these can shape the effectiveness of teaching and learning [7].

## Methods

### Study design

This was a questionnaire-based study, with a particular focus on collecting exploratory qualitative data to tap into educators’ experiences. Quantitative questions were primarily designed to collect demographic and descriptive data related to online teaching experiences post pandemic. Open-ended qualitative questions utilized the Critical Incident Technique (CIT) as a framework for data collection and analysis [11]. The Critical Incident technique has its roots in organizational psychology and is defined as *“a set of procedures for collecting observations of human behaviour in such a way as to facilitate their potential usefulness in solving practical problems and developing broad psychological principles”* [12]. CITs are essentially unusual and memorable events, produced by the way participants look and interpret the significance of a situation; thus any experience teachers encountered in their teaching may be a critical incident and therefore a situation they can reflect upon [13]. This open-ended procedure encouraged the sharing of experiences directed by the research question and provided flexibility of the data collection process [14]. Although the technique is most often used in interviews or focus groups, researchers have also adapted a questionnaire approach as a way of ensuring anonymity and preventing bottlenecks and delays [15].

### Sample

The study was conducted at the Faculty of Basic Medical Sciences, OAU, Ile-Ife, Nigeria. An opportunity sampling strategy was employed and all educators in these Departments who carried out online Teaching of pre-clinical teaching during the Covid-19 period (2019/2020) were invited to participate. A sample of 18 educationalists volunteered to take part, consisting of eight, seven and three participants from the Departments of Anatomy and Cell Biology, Physiological Sciences and Medical Biochemistry, respectively. Most participants were male (*n* = 17) and in the age range 41–50 years.

### Data collection

Data collection was carried out using an online questionnaire via Microsoft Forms. A link to the questionnaire was sent to the institutional email addresses of respondents along with the participant information sheet and consent form. The invitation email highlighted that anyone who had experience of teaching online during the pandemic can take part in the study. Participants provided information such as age, gender, department, and length of time in their educational role. In addition, they were asked to rate their use of remote teaching methods during, and post pandemic. An open-ended question asked them to indicate the reason for why they teach or do not teach virtually post pandemic.

The main part of the questionnaire was qualitative and was based on CIT philosophy. Questions asked participants to reflect on a particular example (incident) of something or someone that enabled or challenged their online teaching. They were asked to describe, in their own words, what happened and how things turned out in as much detail as possible. Structured interviews were conducted with three educators who preferred this option, in which case the questionnaire was used as a template and the interview was structured and transcribed onto the form. The questionnaire was initially piloted with three academic staff at the same faculty.

### Ethical considerations

Permission for the study was obtained from the gatekeeper (Provost, College of Health Sciences, Obafemi Awolowo University). Ethical approval was processed and obtained from the Ethical Committee of Cardiff University School of Medicine. It is important to note that three of the researchers are faculty members at the institution. Thus, potential bias and effect of relationships were addressed using an online questionnaire or structured interview method and literature was used extensively to reflect on preconceptions. Academic supervision was provided throughout (external), and reflexivity and transparency were ensured in the analysis process. Coding and themes were reviewed and checked for their definition by three researchers until agreement was achieved.

### Data analysis

Descriptive data were obtained for demographic characteristics and to capture participants’ rating of their experiences of teaching online. Qualitative CIT data were analyzed using thematic analysis [16]. Codes were assigned to each incident to capture and identify conceptually recurring patterns. All responses associated with a particular code were then categorized into themes, which were reviewed and defined by three researchers.

## Results

Data were analyzed in line with the research objectives, with a focus on examining participants’ experiences of teaching online and their perceptions of enablers and barriers to online teaching. Overall, findings indicated that some medical educators continued to use online teaching methods post-pandemic, and the motivations were varied. Through exploration of enablers and challenges of teaching online during the pandemic, it was possible to highlight some of the lessons learned from these experiences.

### Online teaching post-pandemic

Participants were asked if they continued to teach remotely post pandemic, out of which most said they have (72%). The remaining had fully gone back to the face-to-face mode. The reasons for continuing to use remote teaching were varied. For some, the motivation came from students’ preference. For example, *‘After the pandemic, students don’t want to come for physical classes…they prefer the online method’(P3).* One participant noted that *‘Students demanded for it’ (P13)* and that *‘Students like it’ (P17).* Other comments suggested that teaching online during the pandemic provided an opportunity for change and diversification of teaching using technology. For example, participant 16 noted that teaching online during the pandemic taught him a new way of delivering his lectures: *‘Students on resumption early this year were to take my class, but it could not hold because of the strike action. I then decided to post my lectures online, hoping that they will soon resume, and we will have face to face interactions for explanation’.* One participant noted that ‘*Online teaching seems to be an easier way of reaching (our) students’ (P2).* It is possible that this acted as a catalyst for continuing this practice, as noted by participant 18: *‘The Institution encouraged online teaching for large classes’.*

Educators who abandoned virtual teaching as soon as they were able to fully resume face to face teaching expressed different views. As suggested by participant 9, *‘I thought that the University wants us to discontinue online teaching after the pandemic’.* Another participant made a particular reference to teaching practical classes, which are central for pre-clinical teaching. He noted that he wished to *‘engage the students in full face-to-face practical classes (P15)’.*Practical classes are pivotal to the teaching of pre-clinical subjects and had to be postponed as noted by participants: ‘*Physical classes were conducted after students returned to the campus with students being divided into small groups’ (P1)*, and *‘We brought in students in batches for physical interaction during practical…our practical sessions were not so effective because limited time was available for each batch of students (P18)’.* Whilst most educators agreed that some elements of online teaching worked well, this was not the case with practical classes. *‘…online classes may not replace the practical demonstration and so online teaching has its limitations and shortcomings, … it should not be over romanticized” (P2).*

### Enablers and barriers to online teaching and lessons learned

Five main themes were identified that captured participants’ reflections on their experiences of teaching remotely that can inform future directions. These were: Skill and training, Motivation and attitudes, Internet and connectivity, Learners’ behaviors, and Socio-economic constraints (see Table 1).

**Table 1:** Themes and definitions of enablers and challenges to online teaching

Theme	Definition
1. Skills and training	Previous experience of online teaching and / or benefited from attending training sessions
2. Teachers’ motivation and attitudes	Being motivated to commence online teaching to meet the pressing needs of learners and appreciating the perceived benefits of online teaching
3. Internet and connectivity	Access to internet on the University campus or at home. Situations of power outage, fluctuations in internet service
4. Learners’ behaviors	Conduct or misconduct of learners online, leading to discouragement and frustration
5. Socio-economic constraints	Financial constraints impacted the availability and access to internet and technology

#### Theme 1: skills and training

 This theme highlighted the benefits of having some previous experience of online teaching and the usefulness of training that was available to them. As noted: *’Previous use of zoom for informal activities helped me to adjust to online teaching” (P11).* Similarly, participant 9 commented on how he was able to benefit from his previous experience of teaching ‘*part-time Nursing undergraduate students of the open distance learning programme online for a few years before the pandemic’*. Indeed, even a one-day training event organised by the University was useful, with comments such as ’*Training was organized … and this helped me to handle the classes‘ (P2)*, and *’My understanding of the technology for online teaching improved appreciably after undergoing the training organized by the University‘(P3).* Participants who took advantage of unplanned external and informal training opportunities also noted some benefits, such as *‘At the onset of online real time teaching using Google meet, I was faced with the challenge of 100 space limit … a colleague from AISPI (African Institute for Science Policy and Innovation) sent notice for a workshop on methods by which google meet could be used without limit to the number of participants’* (*P18*). Some also learnt from more experienced colleagues: *‘Colleagues in my Department who are familiar with online methods also assisted me’ (P16).*

#### Theme 2: teachers’ motivation and attitudes

This theme reflects educators’ comments that capture their motivation to continue meeting students’ educational needs. Some educators commented that it was their duty as teachers and *‘it was just a necessity’ (P5*) and that *‘the need to engage students for productivity was the driving force for the online teaching” (P4) so they ‘had to adapt’ (P12).* For others, the online learning environment gave a feeling of safety and convenience. As noted, ‘*It was convenient and safe for both the teacher and the students (P15).’* Similarly, participant 3 said that he ‘*enjoyed the ease and comfort of being able to work remotely’.* For some, it eliminated the time and expenses required for travelling, as noted by participant 7 ‘*It saves me the trouble of going to school whenever I have lectures. I teach in the comfort of my house”.*

#### Theme 3: internet and connectivity

 The availability of resources and internet facilities constituted a major factor to online teaching and learning including issues such as frequent power outage, poor internet service, and shortage of technical support. Educators who were able to access the campus took advantage of the internet services available there. As suggested by one participant: *’The internet facility provided by the university was very strong and stable without extra cost. This enabled me to conduct classes smoothly‘ (P14).* However, the same participant also observed that power outage sometimes made the internet unstable. Frequent power outage and incessant disruptions in internet connection was a major challenge. For example, ‘*Sometimes you may be set for a lecture and light will go off which will also affect the internet connectivity’ (P6).* Participant 9 provided more detail and said that *’Frequent power outages interfered with internet services. Lecture periods were interrupted and sometimes unnecessarily prolonged, thereby making the students lose concentration. Classes that were badly interrupted had to be rescheduled‘.* Participant 4 described: *’During one of the sessions, I was with the students on zoom and sharing some slides, there was power outage, … I tried to connect with my phone, but the service was poor, and I could no longer communicate effectively with the students. The class had to be cancelled.’* The teaching process and time were severely impacted due to loss of connectivity limiting the teaching process. Participant 7 noted that *‘There were challenges of network failure during lectures. One day, I had a lecture at 9am but I could not link up until 9.30am’.* Another participant said that *‘Internet facility was cut off during a lecture due to power outage, thereby disrupting the lecture. I had to restart lecture several times, although I managed to finish up’ (P14).*

This was aggravated by perceptions that they had to work things out for themselves as noted by one participant: ‘…*did not provide adequate technical support to both teachers and students to make learning easy’ (P18).’.* This issue is reflected in comments made by others. For example, participant 2 highlighted *‘hardship and discouragement as a result of internet fluctuations from network provider’.* Participant 12 described a similar incident where *‘There was this time I was having an online class and I ran out of data. The class had to end abruptly. There are times students will complain of not having network to connect to the class or not being able to charge their devices due to power outage.’* Thus, educators were restricted to the use of high immediacy and low bandwidth applications such as whatsApp and telegram, which are restrictive and not specifically designed for educational purposes. As noted by participant 12, ‘*We attempted to use google classroom but there were always internet fluctuations. We had to shelve the idea and settle for WhatsApp platform’.*

#### Theme 4: learners’ behaviors

 This theme is defined by situations in which the behaviors of learners impacted the teaching and learning experiences. Positive behaviors encouraged and motivated teachers to put more effort into their online teaching, whereby negative behaviors had the opposite effect. This reinforces the reciprocal nature of the learning experience. For example, non-attendance and lack of proper comportment created a sense of frustration and discouragement for the educator. For example, participant 1 noted: *‘Students either joined the class late or left without notice. Low turnout was very discouraging’.* Another participant noted that he *‘started a class with full attendance and then students started to leave. This got me demotivated and frustrated. I later took time to express my feelings to them and their attitude changed’ (P13).*

On occasions, negative behaviors during online sessions resulted in termination of classes. *‘On a particular day, students were just so uncontrollable that I had to exit the class. There was so much noise making and this obstructed the smooth flow of the class for the day. Generally, there were too many interruptions from the students, noise making, intermittent disturbances and they could not be controlled, probably because they lacked experience (P5)’.* Participant 8 made a similar comment *‘Some students made rude remarks on the chat page. I was discouraged and had to suspend the class. The class representative later apologized on behalf of the class’.*

When teachers felt that their teaching ‘*worked well’ (P12)*, and when they received positive feedback from learners, they became more motivated to teach. Comments such as *‘students showed preference for online teaching‘ (P7)*, and *’Students responded promptly to questions and submitted assignments on time‘ (P11).* For some, motivation came from students’ requests and desire to be engaged online as they were getting fed up with staying idle. As noted by participant 8, ‘*Students requested for it based on their inability to convey in their physical classrooms’.* For others, receiving positive feedback from students was a motivating factor, which encouraged them to put more efforts into preparing for the classes. As noted by participant 13, ‘s*tudents informed me that they were benefitting a lot from the online classes as it inspired them to learn more on their own. This made me decide to put in more efforts’.*

#### Theme 5: socio-economic constraints

 This theme reflects comments made by educators related to theirs as well as learners’ financial constraints and lack of access to data and technology. Many educators received complaints from learners that they could not afford to participate in zoom lectures or access uploaded videos because of the large volume of data required. Some educators also complained about out-of-pocket spending to purchase data or repair their faulty devices. As pointed out by participant 2, ‘*There was a lot of out-of-pocket spending as I tried to find an alternative source of internet service’* and *‘many could not afford using their personal mobile data for zoom or Google meet and many educators merely dumped materials on google classroom for students to read (P18).* Having to look for alternative internet services impacted the teaching and learning experience. ‘*Some students are unable to buy data. This reduced number of participants in class. Many students and teachers could not afford to use their personal data for zoom or google meet teachings’ (P15).* The impact on teaching was also noted by participant 3 who said that he *‘missed one or two classes because my device misbehaved. It was not a palatable experience. I had to apologize to the students. Lecturers and especially students who do not have electronic devices due to economic factor cannot participate in online class’.*

## Discussion

The aim of the study was to examine educators’ experiences of teaching online during- and post-pandemic to inform future institutional directions regarding the teaching of pre-clinical subjects. Findings revealed that educationalists who teach pre-clinical subjects would like for didactic based content to be available online, but for practical classes to be conducted in-person. Educators highlighted that both them and students benefit from the flexibility and convenience of online learning and that greater clarity of the institutional strategy regarding online teaching would be beneficial [17]. To this end, findings suggest that OAU would benefit from reflecting on lessons learned with particular focus on skills and training of teachers; teachers’ motivation and attitudes; internet and connectivity, learners’ behaviors, and socio-economic constraints.

Findings provide further evidence indicating that if internet services are inadequate and the technology available for teachers and learners is limited, they are unable to use advanced education-oriented platforms [18, 19]. When teaching is described as unpleasant by the teacher, it carries with it inevitable consequences as learners can become unruly and disorganized when they are not satisfied with the conditions of learning [18]. Poor attitudes of learners have been identified as a challenge to online teaching in this study and was likely to have demotivated the teacher and reduced teaching effectiveness, as was evidenced in previous researches [19–21]. This implies that addressing the technological challenges is imperative and must be considered within the socio-economic constraints that are unique to resource constrained institutions. The introduction of a Learning Management System is an important first step and learning from the experiences of other LMICs, sharing resources with other institutions for example, can go some way towards overcoming financial constraints [22].

Findings add to an increasing body of literature evidencing that online teaching poses a challenge to teachers primarily trained to teach face-to-face. More specifically, educators with prior experience and knowledge of ICT found it easier to migrate to online teaching during the pandemic [19]. This highlights teachers’ need to upskill and adapt to new pedagogical concepts and modes of teaching delivery [20]. Increasing feelings of confidence and motivation amongst teachers will likely have a positive impact on students’ online learning experience and students will be more likely to embrace this mode of learning [19, 23]. A review conducted by Pettersson highlighted that *‘students need competent and confident teachers to make use of rich digital learning environments’* [24]. Studies have shown that training and perception of proficiency helps educators to develop confidence to engage learners and this impacts on performance, satisfaction and outcome [23]. However, in comparison to institutions based in High-Income countries, higher education institutions such as OAU are likely to have less resources and funds available for training and development of their teaching staff [5]. In addition, the unstable and inconsistent access to the internet suggests that synchronous based teaching activities may be a disadvantage to learners. Indeed, remote teaching and learning in resource- constrained countries is primarily supplemental to provide content offline so it can be accessed at a time and place convenient to learners [25, 26]. With this in mind, it is important that training and development of teachers takes such constraints into consideration and provides teachers with the tools and skills that enable them to operate successfully in a blended learning teaching environment suitable for their context. Cost- effectiveness should be a guiding principle, for example, focusing on digital teaching methods that do not require a substantial amount of data to download, with a focus on asynchronous activities [25].

It is important to note that although the study provided useful insights for the institution and contributes to the emerging body of literature on lessons learned from teaching during the Covid-19 pandemic, it is not without limitations. To begin with, the study was context specific and only considered the experiences of teachers of pre-clinical subjects. Further research may be conducted to ascertain whether the findings are applicable to other academic subjects. The relatively small sample size and the imbalance of male to female ratio are also a potential limitation. However, the sample did include a good representation from the different pre-clinical subjects taught at this specific setting and most educators of pre-clinical subjects in this institution are male. Worthy of note is also the potential limitation of the CIT methodology that relies heavily on people’s memory of incidents [14] and the potential risk to credibility of data in qualitative research [27]. It can be argued however that qualitative researchers *‘are an integral part of the process and final product, and separation from this is neither possible nor desirable’* [28].

## Conclusion

The overarching purpose of the present study was to examine educators’ experiences of teaching online during and post-pandemic to inform the development of a blended learning approach for teaching pre-clinical subjects. Findings provide further evidence that the pandemic opened opportunities for innovations and that the successful implementation of a blended strategy requires careful consideration of financial, institutional support, technological and individual factors [25–29]. Institutional commitment and clarity of an educational strategy is at the heart of such change and the use of a Learning Management System is required to host teaching and learning activities. In addition, faculty and students should be equipped with the necessary skills and provided with the resources they need to operate in this environment is imperative. Further evaluation may be conducted as a follow-up to examine whether the blended learning strategy achieved its objectives and whether the recommendations made were implemented successfully.

## Data Availability

All data are stored at Cardiff University in compliance with the University Research Records Retention Regulations and can be made available upon request to the corresponding author.
